# Biological Control Potential and Drawbacks of Three Zoophytophagous Mirid Predators against *Bemisia tabaci* in the United States

**DOI:** 10.3390/insects11100670

**Published:** 2020-10-01

**Authors:** Amy Roda, Jose Castillo, Carina Allen, Alberto Urbaneja, Meritxell Pérez-Hedo, Scott Weihman, Philip A. Stansly

**Affiliations:** 1 United States Department of Agriculture, Animal Plant Health Inspection Service, Plant Protection and Quarantine, Science and Technology, Miami, FL 33158, USA; carina.l.allen@usda.gov (C.A.); scott.w.weihman@usda.gov (S.W.); 2 Department of Entomology and Nematology, Southwest Florida Research and Education Center, University of Florida, Immokalee, FL 34142, USA; castillojos@hotmail.com (J.C.); pstansly@ufl.edu (P.A.S.); 3 Instituto Valenciano de Investigaciones Agrarias (IVIA), Centro de Protección Vegetal y Biotecnología, Unidad de Entomología, Carretera CV-315, Km 10′7, 46113 Moncada, Spain; aurbaneja@ivia.es (A.U.); meritxell_p@hotmail.com (M.P.-H.)

**Keywords:** biocontrol, mirids, herbivory, whitefly, invasive pests, Integrated Pest Management

## Abstract

**Simple Summary:**

The silverleaf whitefly, *Bemisia tabaci*, is a serious economic pest of tomatoes, particularly as this insect can carry devastating plant diseases. Growers currently rely on costly insecticides and biocontrol agents may offer a viable alternative in the integrated pest management of tomatoes. We studied one established and two native omnivorous plant bugs’ (mirids) ability to control whiteflies, whether they damaged tomato plants, and their ability to persist in the crop. Established biocontrol agents have advantages as they typically have little impact on non-target native species, they have adapted to the local environment and are less expensive than importing and testing exotic agents. In field cage studies, all three species controlled whiteflies. However, the damage the mirids caused to tomato plants varied greatly. We also tested whether an alternate host plant, sesame, could increase mirid numbers and reduce plant damage. These experiments showed that the benefits of sesame varied among the mirid species. Although not all established generalist mirids would be suited for use as biocontrol agents, this study showed that two of USA’s mirid species could be immediately available to help manage existing and future invasive pests of tomato.

**Abstract:**

Miridae (Hemiptera) of the tribe Dicyphini are important zoophytophagous predators use to control pest arthropods in vegetable crops. However, the risk that their herbivory may cause economic damage could hinder their application as useful biocontrol agents and may limit the likelihood they would meet regulatory requirements for importation. We conducted field cage studies to assess the predation capacity and tomato plant damage of three mirid species established in south USA, a known biocontrol agent (*Nesidiocoris tenuis*), and two native species (*Macrolophus praeclarus* and *Engytatus modestus*). All three species significantly reduced the number of whiteflies (*Bemisia tabaci*) on tomato plants compared to tomato plants without mirids. More damage, evaluated as the number of necrotic rings, was observed on tomato plants with *E. modestus* and *N. tenuis* compared to *M. praeclarus.* In our experiments that included sesame plants (*Sesamum indicum*) with tomato plants, mirid numbers increased despite a low number of prey, thus showing a benefit of the plant-feeding habit of these predators. USA’s established mirids may therefore prove to be immediately available biological agents for the management of present and future tomato pests.

## 1. Introduction

Plant feeding predators are useful biocontrol agents in many cultivated crops [1,2,3]. Their unique benefit lies in their ability to persist in the crop in the absence of prey as they can survive and reproduce on non-pest food sources, such as pollen, nectar or other plant material of cultivated and wild origin. However, the benefits in controlling pest species may be counteracted by the economic damage they may cause as herbivores [4,5,6].

Omnivore predators display a range of carnivorous and herbivorous feeding behavior. Some arthropods are opportunistic omnivores, and they reside on a continuum between phytozoophagy (prey-taking herbivores) and zoophytophagy (plant-feeding predators) [7,8]. For biocontrol, an optimal agent would actively seek the pest as prey and feed minimally on the crop plant [9]. However, a preference for prey may cause the biological control agent to leave the crop when prey numbers are low. Their migration from the crop could be reduced by the presence of preferred non-crop plants offering better resources to the plant-feeding predator. The non-crop plant could also be utilized to minimize feeding damage to the crop [3].

Zoophytophagous mirid predators are used in several cropping systems in combination with selective pesticides [2,3]. In Europe, *Macrolophus pygmaeus* (Rambur), *Nesidiocoris tenuis* (Reuter)*, Dicyphus bolivari* (Lindberg) and *Dicyphus errans* (Wolff) (Hemiptera: Miridae) are the main generalist predators of tomato pests [1,10,11,12], although only *M. pygmaeus* and *N. tenuis* are commercially available and widely used. *M. pygmaeus* is mainly used in production areas with temperate climates, while *N. tenuis* is used in areas with warmer climates [3]. In North America, the native species, *Dicyphus hesperus* Knight, is commercially available and is used in temperate areas of Canada, Northern USA, and recently in Mexico to control several pests of greenhouse grown tomatoes [3,13,14,15]. Nevertheless, plant bugs may also damage tomato plants, especially when prey is scarce and dispersal is limited by confinement in greenhouses [4,5,16]. The injury caused by different species varies and is related to the number of pests found on the crop. The risk that their herbivory may cause economic damage at times hinders their acceptance as useful biocontrol agents and may limit the likelihood they would meet regulatory requirements for importation to new areas/countries [4,17]. Therefore, utilizing native and established zoophytophagous predators may provide an alternative to importation of exotic species [3,15].

There are multiple benefits of using locally occurring zoophytophagous mirids. They typically have little impact on non-target native species that have coexisted with the control agent. They have adapted to the local environment and can persist in the environment on alternative prey and hosts. Importantly, there is a substantial savings in time and expense of not importing a new biocontrol agent, i.e., foreign exploration, evaluation in quarantine, and undergoing the permitting process for releases in the open fields. However, there are potential issues with using generalist biocontrol agents in a new pest and host system. They may not show strong preference for an introduced pest species, may not be adapted to the pest’s (crop) environment, and there may be a delay in the time to recognize or find the new prey. In addition, zoophytophagous mirids may show more preference for the crop than the pest. Controlled evaluation is therefore prudent before releasing the agent into a cropping system [18].

In this study, we explored the potential use of three mirid species found in the USA to control the sweet potato whitefly, *Bemisia tabaci* (Gennadius) (Hemiptera: Aleyrodidae) in greenhouse and field cultivated tomato. This important crop is under constant threat from *B. tabaci* (Middle East-Asia Minor 1 (MEAM1) biotype (formerly known as Biotype B) largely due to its role as a virus vector [19]. More recently a sibling species, Mediterranean (MED) *B. tabaci*, has invaded the USA and is distinguished by its ability to rapidly evolve resistance to key insecticides [20]. Pesticides can provide some relief from these pests, but alternatives are needed because of pesticide resistance, environmental issues, health concerns for workers and consumers, and prohibitive costs [21]. We tested the ability of one widely distributed species (*N. tenuis*), and two native mirids (*Macrolophus praeclarus* (Distant) and *Engytatus modestus* (Distant)) to control *B. tabaci* on tomato in field cage studies. *N. tenuis* is extensively used as a biological control agent for whiteflies and lepidopterans in Southern European tomato production systems [1]. Although *N. tenuis* is considered one of the most effective zoophytophagous biocontrol agents, this mirid can become a pest of tomato particularly in mild climate regions [22,23]. *N. tenuis* feeding produces necrotic rings around stems as well as damage to flowers and fruits to a level that can cause economic loss [4,24,25,26,27]. To reduce the negative impact of *N. tenuis* phytophagy, management strategies have been explored, including selective pesticide applications, addition of supplemental food and companion planting with sesame (*Sesamum indicum* L. (Lamiales; Pedaliaceae)) [5,28]. *E. modestus* is considered a pest species in tomato and tobacco [29,30]; however, this mirid has been documented to feed on pest species such as aphids, mealybugs, psyllids, leafminers, lepidopteran (*Helicoverpa* spp.) eggs and early instar larvae [31,32,33]. Similar to *N. tenuis*, *E. modestus* produces necrotic rings and damages the tomato plants to a level in which economical loss occurs [29]. In contrast, *M. praeclarus* has not been reported as a pest although found on tomato and tobacco [34,35]. This mirid is considered a predator of multiple Solanaceae pests including *B. tabaci*, the tobacco budworm *Heliothis virescens* (Fab.) (Lepidoptera: Noctuidae), the green peach aphid *Myzus persicae* (Sulzer) (Hemiptera: Aphididae), and the onion thrips *Thrips tabaci* Lindeman (Thysanoptera. Thripidae) [36,37]. Studies on *M. praeclarus* showed that the mirid did not cause feeding damage to tomato plants compared to *N. tenuis* and an analysis of its predation capacity and thermal requirements suggests that the mirid may have a role as a biocontrol agent in temperate climates [38]. In addition to determining whether these three mirid species controlled *B. tabaci*, we compared their population increase and how much each species damaged tomato plants. We also determined whether adding the companion plant, sesame, as an alternate food source, affected mirid numbers, damage to tomato plants, and their ability to control whiteflies. This information will be important in determining whether these mirids will be effective and safe biocontrol agents for use in cultivated tomato production systems.

## 2. Materials and Methods

### 2.1. Plants and Colonies

Tomato (cv. Florida 91, Tomato Growers Supply Company, Ft. Myers, FL, USA) and sesame plants were grown from seeds in controlled environmental chambers (25 ± 1 °C; 70 ± 15 RH; 16:8 h L:D). A commercial potting mix (PRO-MIX HP Mycorrhizae, Premier Tech Horticulture, Quakertown, PA, USA) was used and fertilizer (Miracle-Gro^®^ Water Soluble All Purpose Plant Food, ScottsMiracleGro, Marysville, OH, USA) applied every 14 days. The plants were seeded 4–5 weeks before use in the colonies or the experiments. Tomato plants, approximately 30 cm high, were introduced into insect rearing cages (Bugdorm MegaView Science Company, Ltd., Taichung, Taiwan) as needed. *B. tabaci* and *E. modestus* were collected from an abandoned tomato field in Collier County, FL, USA. *N. tenuis* and *M. praeclarus* were collected from ornamental succulent sesame *Uncarina grandidieri* (Baill.) Stapf (Lamiales: Pedaliaceae) plants located in Miami–Dade County, FL, USA. Voucher specimens of the three mirid species are held at the Florida Department of Consumer Services, Florida State Collection of Arthropods, Gainesville, FL, USA. Collected mirids and whiteflies were reared on pesticide-free tomato seedlings. Mirids were fed with a mix of frozen eggs of *Ephestia kuehniella* Zeller (Lepidoptera: Pyralidae) and cysts of the crustacean *Artemia* spp. (Anostraca: Artemiidae) (1:5 ratio respectively (Koppert Biological System, Howell, MI, USA) offered *ad libitum* on the adhesive portion of repositionable notes (Post-it^®^ Brand, 3M Cynthiana, KY, USA). Mirid adults and whiteflies were collected using a mechanical aspirator (Hausherr’s Machine Works, Toms River, NJ, USA).

### 2.2. Evaluation of Established and Native Mirids

Cage studies were conducted to determine whether *N. tenuis, M. praeclarus,* and *E. modestus* could control *B. tabaci* populations and whether feeding damage on tomato plants differed amongst the three mirid species. The experiments were conducted inside three portable field cages (183 × 366 × 183 cm with 32 × 32 *Lumite*^®^ mesh screen (BioQuip Products, Inc. Rancho Dominquez, CA, USA) located at the United States Department of Agriculture (USDA), Subtropical Horticulture Research Station (SHRS), Miami, FL (25.643464°, −80.295132°, World Geodetic System (WGS) 84). Insect rearing cages 93 × 47.5 × 47.5 cm (BugDorm; MegaView Science Co., Ltd.; Taichung, Taiwan) were placed on benches inside the field cages and a piece of plexiglass was placed on the top of each insect rearing cage to prevent rain-water from entering. Each field cage held 2 complete replicates of each treatment. Temperature and humidity were recorded continuously using environmental data loggers (HOBO^®^ Onset Computer Corporation Bourne, MA, USA) and obtained from local weather stations (KFLPINEC3, Weather Underground, The Weather Company, LCC, Figures S1 and S2). The daily temperature and humidity experiment averaged 25.48 ± 0.28 °C (range 24.3 to 27.6 °C) and 75.97 ± 0.86% RH (range 67 to 75% RH) respectively. Five tomato plants were placed in each insect rearing cage and watered daily using drip irrigation placed into each pot. Two experiments, each with four treatments, were conducted. The treatments were (1) a no insect control (tomato plants only), (2) whiteflies only, (3) whiteflies with *M. praeclarus,* and (4a) whiteflies with either *E. modestus* (experiment 1 conducted April 24–June 5, 2015, n = 5) or (4b) whiteflies with *N. tenuis* (experiment 2, conducted March 24–May 4, 2017, n = 6). Fifty adult whiteflies (10 per plant) were aspirated from the colony into five vials, and these vials were equally distributed in the whitefly treatment cages prior to releasing the whiteflies. After 10 days, five males and five female mirids (1 pair/plant) were randomly collected from the colony and released into the mirid treatments. To achieve the duel goal of assessing the mirids ability to control whiteflies and to accurately count the total number of the very cryptic and highly mobile adult mirids and nymphs, a single plant was removed and destructively sampled each week. The first plant was removed 1 week after releasing the mirids. The height of the tomato plant and the number of flowers were recorded. Mirid damage was assessed by counting the number of necrotic rings found on the plants. After the damage assessment, all nymph and adult mirids and whiteflies were counted and removed. The plants were then cut in pieces (approximately 4–5 cm), placed in Petri dishes (9 cm in diameter) then held in environmental chambers (25 ± 1 °C; 60% ± 10 RH; 16:8 h L:D) in order to allow the development of mirid eggs [39]. Two weeks later, the number of nymphs were counted to determine the total number of nymphs (the sum of both counts).

### 2.3. Effect of Adding Sesame on Mirid Population Growth and Damage

Cage studies were conducted to determine whether adding a sesame plant could increase mirid numbers in the absence of prey. Only two of the species, *N. tenuis* and *M. praeclarus* were used in the study. *E. modestus* was excluded because the proportionally higher amount of damage and unlikely acceptance as a biological control agent. As described above, insect rearing cages 93 × 47.5 × 47.5 cm were placed on benches inside the field cages. Plants and mirids were added to each rearing cage with one of four treatments (n = 8): (1) *M. praeclarus* with two tomato plants, (2) *M. praeclarus* with one tomato and one sesame plant, (3) *N. tenuis* with two tomato plants and (4) *N. tenuis* with one tomato and one sesame plant. Two female and two male *M. praeclarus* or *N. tenuis* were introduced. The insect rearing cages were held in the field cage for 6 weeks (16 October–27 November 2018) and environmental conditions were monitored. Each field cage held 2 complete replicates of each treatment. After 5 weeks, the plants were removed, heights measured, and the number of mirid feeding rings and flowers found on the tomato plants were counted. All nymphs and adult mirids found on each plant species were counted. As described above, the plants were cut into pieces and held in environmental chambers for two weeks to allow development of eggs. The sum of nymph counts was used to assess mirid population growth.

To determine whether adding a sesame plant reduced mirid feeding damage to tomato plants in the presence of *B. tabaci*, a final cage study was conducted in the outdoor field cages as previously described. This study consisted of six treatments (n = 6): (1) Tomato plants alone with no insects (control for plant height and number of flowers), (2) tomato plants alone with whiteflies, (3) *M. praeclarus* + whiteflies with tomato only, (4) *N. tenuis* + whiteflies with tomato only, (5) *M. praeclarus* + whiteflies with tomato and sesame, and (6) *N. tenuis* + whiteflies with both tomato and sesame. Five tomato plants were added to each insect rearing cage and one sesame plant was included for the tomato and sesame treatments. Fifty adult whiteflies were released into each treatment with whiteflies. After 10 days, five males and five female mirids were added to the mirid treatments. Each week following the mirid release, one tomato plant per cage was removed. The height of the tomato plants was measured and the number of flowers were counted. Mirid feeding damage was assessed by counting the number of necrotic rings found on the tomato plants. All nymph and adult mirids and whiteflies were counted and removed. On week five, the last tomato plant and sesame plant were removed and damage and the number of whiteflies and nymphs was assessed. As previously described, all plants were cut in pieces, placed individually into petri dishes and then held in environmental chambers for two weeks in order to allow the development of mirid eggs. The number of nymphs were counted to determine the total number of mirids.

### 2.4. Statistical Analyses

Insects count data were tested for normality (Shapiro–Wilk test) and homoscedasticity (Bartlett test) using software from SAS Institute (JMP, Cary, NC, USA) [40]. The total number of adults and nymphs of the three species of mirids and the whiteflies were transformed using log (x + 1) to meet the assumptions of analysis of variance (ANOVA). For the study that compared mirid population growth in the absence of prey, the data did not meet assumptions of ANOVA after transformation, therefore, the data were analyzed with non-parametric tests (Mann–Whitney) [41]. The data for all other experiments were analyzed using a two-factor nested random effects model with treatment and week sampled considered fixed factors and rearing cage as a random factor. Treatment was nested within rearing cage. No insects were observed in the no insect control treatment (no whiteflies and no mirids) and the treatment was removed from the analysis of insect numbers. Pairwise comparisons of the fixed factor levels were made using the Tukey’s HSD post hoc tests. *p*-values ≤ 0.05 were considered statistically significant.

## 3. Results

### 3.1. Evaluation of Established and Native Mirids

#### 3.1.1. Whitefly Control by Established Mirids

**Figure 1:**
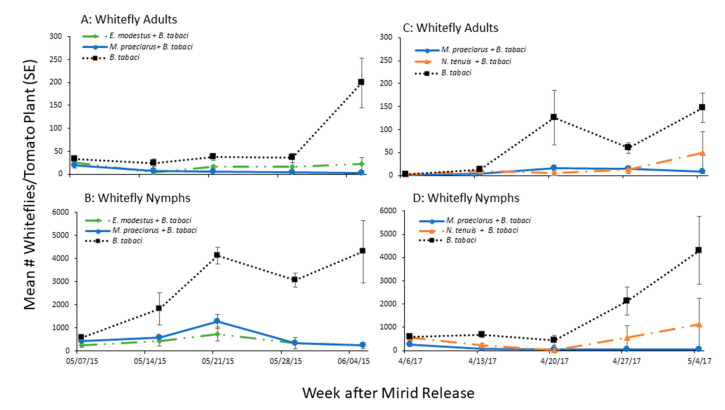
Mean (±SE) number of adult and nymph whiteflies (*Bemisia tabaci*) found on tomato plants in two 5 week field cage studies with three different mirid predators. Experiment 1 (**A**,**B**) compared whitefly population growth in cages with no predators (*B. tabaci*) and with predators *Macrolophus praeclarus* (*M. praeclarus* + *B. tabaci*) or *Engyatus modestus* (*E. modestus* + *B. tabaci*). Experiment 2 (**C**,**D**) compared whitefly population growth in cages with no predators (*B. tabaci*) and with predators *Macrolophus praeclarus* (*M. praeclarus* + *B. tabaci*) or *Nesidiocoris tenuis* (*N. tenuis* + *B. tabaci*).

Mirids were found to reduce the number of whiteflies in the two cage studies (Figure 1, Table 1). In the first experiment, *M. praeclarus* significantly reduced the total number of whiteflies by 100% and *E. modestus* significantly reduced their number by 94% compared to the treatment with no mirids six weeks after adding the mirids. *M. praeclarus* reduced whitefly numbers significantly sooner than *E. modestus* (Figure 1, Table 1). When each week was analyzed separately, post hoc comparisons showed there were significantly fewer total number of whiteflies 4 to 6 weeks after *M. praeclarus* was added and 5 to 6 weeks after *E. modestus* was added compared to the whitefly treatment only (Figure 1A,B). In the second experiment, *M. praeclarus* and *N. tenuis* reduced whitefly numbers by 99 and 81%, respectively 6 weeks after adding the mirids compared to the treatment without mirids (Figure 1C,D, Table 1). *M. praeclarus* reduced the number of whitefly nymphs earlier than *N. tenuis* (Figure 1C,D, Table 1). Post hoc comparisons using Tukey’s HSD test showed there were significantly fewer whiteflies 2 and 3 weeks after *M. praeclarus* was added compared to *N. tenuis*. There were no differences in the number of whitefly nymphs between the two species 4 to 6 weeks after adding the mirids.

#### 3.1.2. Mirid Population Growth and Plant Damage

**Figure 2:**
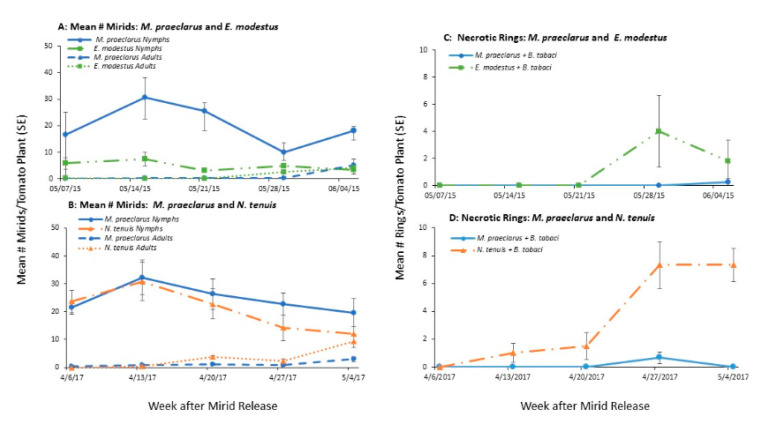
Mean (±SE) number adult and nymph mirids and the damaged caused by the mirids (necrotic rings) on tomato plants inoculated with whiteflies (*Bemisia tabaci*) in two five week field cage studies. Experiment 1 (**A**,**C**) compared mirid population growth and damage of *Macrolophus praeclarus* and *Engytatus modestus*. Experiment 2 (**B**,**D**) compares mirid population growth and damage of *Macrolophus praeclarus* and *Nesidiocoris tenuis*.

Mirid population growth in the presence of whiteflies varied among the species (Figure 2, Table 1). The number of *E. modestus* remained constant throughout the course of the study and there were significantly more *M. praeclarus* on tomato plants with whiteflies compared to *E. modestus* (Figure 2A, Table 1). In both experiments, *M. praeclarus* numbers increased during the first weeks of the study when whitefly numbers were at their peak. As the number of whiteflies decreased, so did the number of mirids found on the tomato plants (Figure 2A,B). *N. tenuis* numbers showed a similar population growth pattern as *M. praeclarus*. They increased for two weeks after adding the adults and decreased the subsequent three weeks. There were no differences in the number of *M. praeclarus* and *N. tenuis* found on tomato plants with whiteflies (Figure 2B, Table 1).

The amount of damage, seen with the number of necrotic rings, varied among the mirid species (Figure 2, Table 1). Although there were fewer *E. modestus* compared to *M. praeclarus*, the number of rings per tomato plant was higher (Figure 2C). Rings were not observed on tomato plants with *M. praeclarus* until six weeks after adding the mirids. Also, these rings were both more pale and thin compared to rings caused by *E. modestus* and *N. tenuis*. Necrotic rings were seen the 5th and 6th weeks after adding *E. modestus* to the tomato plants and their number ranged from 0–14 and 0–8 per plant, respectively (Figure 2C). The tomato plants in cages where *E. modestus* was added were 35% shorter and had 75% less flowers compared to the plants with no insects (Figure S3A,C). In contrast, the tomato plants with *M. praeclarus* were similar in height and had 45% more flowers compared to the tomato plants with no insects (Figure S3A,C).

*N. tenuis* caused significantly more necrotic rings and the rings appeared earlier compared to *M. praeclarus* (Figure 2D, Table 1). Necrotic rings on tomato plants with *M. praeclarus* were seen the 5th week after adding the mirids only and their number ranged from 0–2 rings per plant. The tomato plants with *N. tenuis* had visible rings by the 3rd week after adding the mirids. By the 5th week all tomato plants in cages with *N. tenuis* had necrotic rings and their number ranged from 2–12 per plant. The plant height and the number of flowers was similar for both mirid species, which was not different from plants without whiteflies (Figure S3B,D).

### 3.2. Effect of Adding Sesame on Mirid Population Growth and Plant Damage

**Figure 3:**
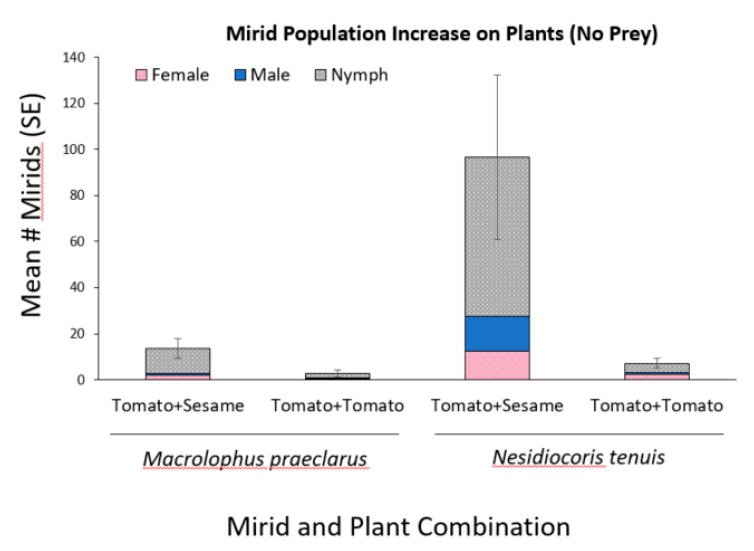
Mean (±SE) number of *Macrolophus praeclarus* and *Nesidiocoris tenuis* nymphs, males and females found in cages with two clean (no whitefly prey added) tomato plants or one tomato and one sesame plant five weeks after adding four adult mirids (two males and two females).

When *M. praeclarus* and *N. tenuis* were provided plants as their sole food source, the number of mirids increased when sesame was present (Figure 3). There was a 13.5 fold increase in the number of *N. tenuis* and a 5.1 fold increase in the number of *M. praeclarus* when sesame was combined with tomato plants compared to tomato plants alone. There were significantly more *N. tenuis* compared to *M. praeclarus* when sesame plants were present (Figure 3, z = 2.92, *p* = 0.004). There was no differences in the number of *N. tenuis* and *M. praeclarus* when only tomato plants were present (Figure 3, z = 1.04, *p* = 0.299). Additionally, *N. tenuis* nymphs were able to complete development when sesame plants were present (Figure 3). There was a 6 and 8 fold increase in the number of female and male *N. tenuis,* respectively, when a sesame plant was present.

**Figure 4:**
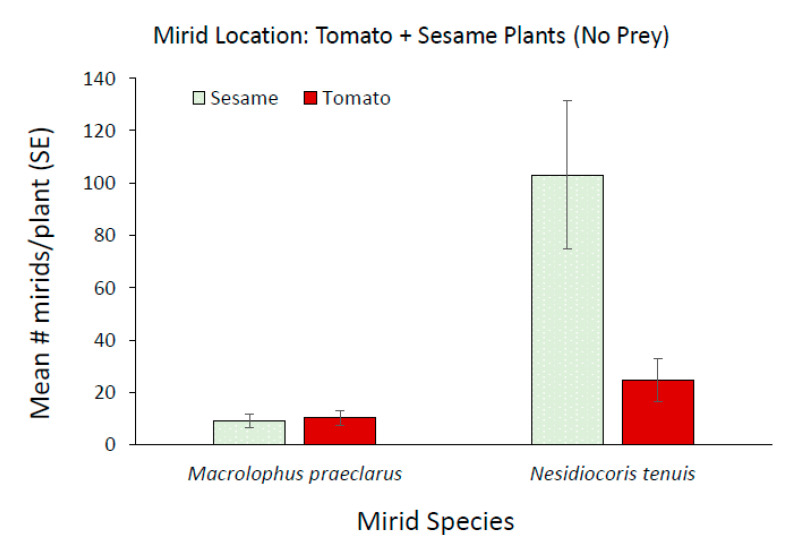
Mean (±SE) number of *Macrolophus praeclarus* and *Nesidiocoris tenuis* adults and nymphs found on clean (no whitefly prey added) tomato and sesame plants five weeks after adding four adult mirids (two males and two females).

Although, *M. praeclarus* nymphs did not complete development when sesame was present during the six week experiment, the plant increased the persistence of the adults. A mean of 2.6 *M. praeclarus* adults from the original four added adults were recovered when sesame plants were present and a mean of only 0.66 adults were recovered from tomato plants alone treatment. Additionally, significantly more *N. tenuis* were found on sesame compared to tomato plants (Figure 4, z = 2.91, *p* = 0.003). In contrast, a similar number of *M. praeclarus* was found on tomato and sesame (Figure 4, z = 1.00, *p* = 0.317). No rings were found on tomato plants when *M. praeclarus* was added to either treatment. In contrast, tomato plants had 8.4 ± 3.15 and 17.25 ± 6.8 (mean ± SE) rings per plant when *N. tenuis* was added to tomato plants paired with another tomato or paired with a sesame plant, respectively. Although there were significantly more *N. tenuis* when a sesame plant was present (Figure 3), there were no differences in the number of rings or plant height of tomato plants when a tomato plant was paired with sesame or when it was paired with another tomato plant (z = 1.04, *p* = 0.27, z = 1.16, *p* = 0.25, respectively). However, the higher number of *N. tenuis* in cages with sesame plants resulted in a 76% decrease in the number of flowers, which was significantly lower compared to cages with only tomato plants (z = 2.09, *p* = 0.04). There was a similar number of flowers (z = 1.84, *p* = 0.06) and there were no differences in plant height (z = 1.09, *p* = 0.27) in cages with *M. praeclarus* with a tomato plant paired with a sesame plant or tomato plant paired with another tomato plant.

### 3.3. Effect of Adding Sesame on Mirid Control of Whiteflies on Tomato

**Figure 5:**
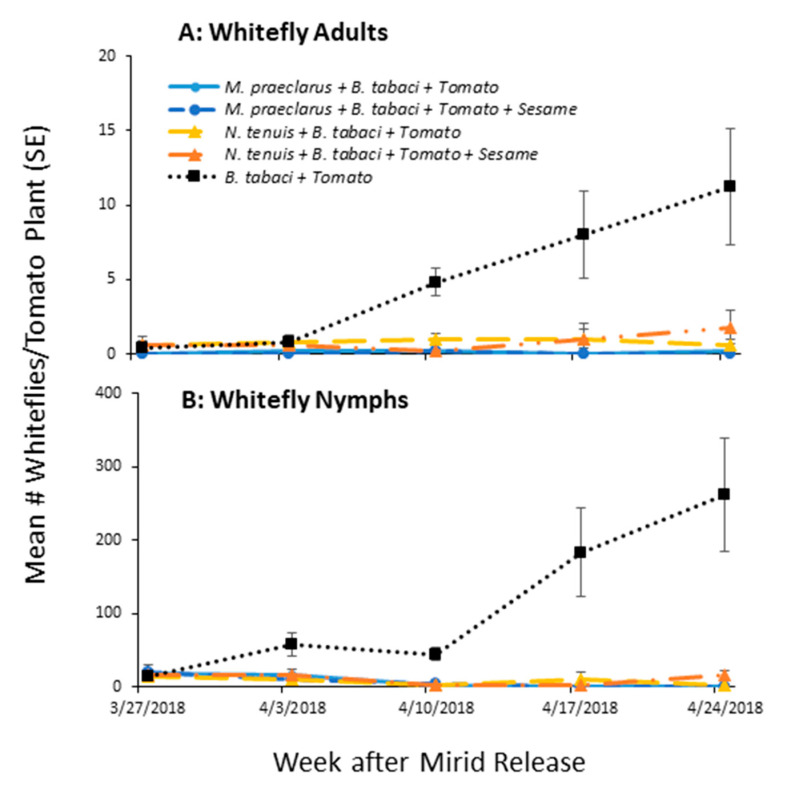
Mean (±SE) number of adult (**A**) and nymph (**B**) whiteflies (*Bemisia tabaci*) found on tomato plants with no predators (*B. tabaci* + Tomato), on whitefly infested tomato plants with *Macrolophus praeclarus* in cages with only tomato plants (*Macrolophus praeclarus* + Whiteflies + Tomato) or 5 tomato plants with one sesame plant added (*M. praeclarus* + Whiteflies + Tomato + Sesame) and on prey infested tomato plants with *Nesidiocoris tenuis* in cages with only tomato plants (*N. tenuis* + Whiteflies + Tomato) or 5 tomato plants with one sesame plant added (*N. tenuis* + Whiteflies + Tomato + Sesame) over a five week field cage study.

Mirids were found to reduce the total number of whiteflies found on tomato plants both in the presence and the absence of a sesame plant (Figure 5, Table 1). *N. tenuis* and *M. praeclarus* reduced whitefly numbers to similar levels on tomato (no differences Tukey’s HSD post hoc comparison). In contrast, there were more whiteflies found on sesame plants in cages with *N. tenuis* (21.6 ± 9.56) than in cages with *M. praeclarus* (0.8 ± 0.58). The presence of sesame did not affect the total number of mirids found on tomato plants in the whole model analysis (Table 1). However, there was a significant interaction between treatment and sample date (Table 1). To better understand the influence of sesame plants on mirid population growth, each species was analyzed separately.

**Figure 6:**
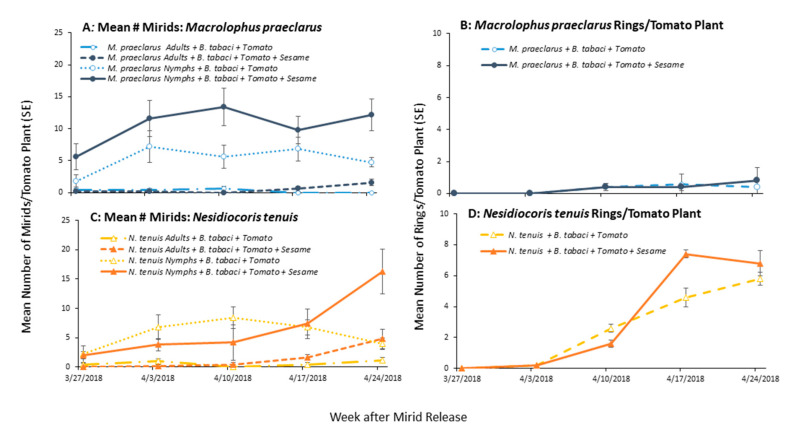
Mean (±SE) number of *Macrolophus praeclarus* (**A**,**B**) and *Nesidiocoris tenuis* (**C**,**D**) mirids and mirid damage (rings) found on tomato plants infested with whiteflies (*Bemisia tabaci*) with and without a sesame plant present over a five week field cage study.

**Figure 7:**
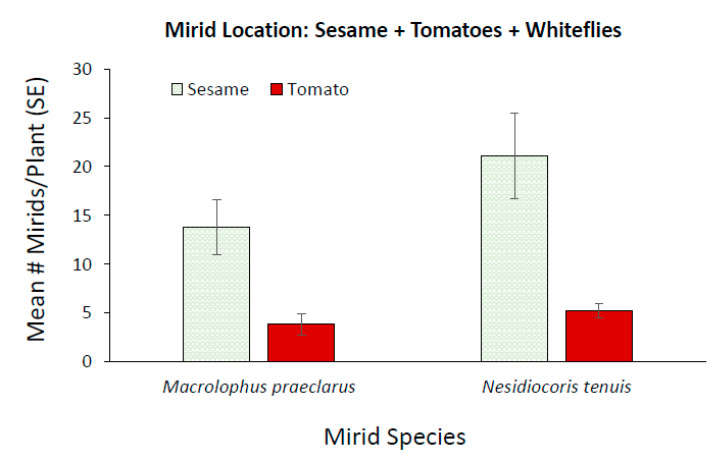
Mean (±SE) number of *Macrolophus praeclarus* and *Nesidiocoris tenuis* adults and nymphs found on whitefly infested tomato and sesame plants 5 weeks after adding adult mirids to field cages.

The presence of sesame with tomato plants significantly increased the total number of *M. praeclarus* found on tomato plants compared to the number found when only tomato plants were present (Figure 6A, Table 1). The number of *M. praeclarus* increased significantly over the course of the experiment (Figure 6A, Table 1). In cages that had both tomato and sesame plants, there were more mirids on sesame plants than tomato plants (Figure 7, z = 3.13, *p* = 0.002). Although there was a 92% increase in the number of *M. praeclarus* found on tomato plants in cages with sesame, there was no significant increase in the number of feeding rings (Figure 6B, Table 1).

For *N. tenuis*, there were no differences found in the total number of mirids on tomato plants with or without a sesame plant present until five weeks after adding the mirids (Figure 6C; Table 1). The number of *N. tenuis* mirids found on tomato plants continuously increased in cages where sesame was present. In contrast, *N. tenuis* numbers remained relatively the same for cages with only tomato plants. In cages that had both tomato and sesame plants, there were more mirids on sesame plants than tomato plants (Figure 7, z = 3.13, *p* = 0.002). *N. tenuis* nymphs reached the adult stage when sesame plants were present and there were twice as many adults recovered than added to the cages. In contrast, only half the number of adults added were recovered from cages with only tomato plants. The number of rings increased significantly over the course of the study and there were no differences in the number of rings on tomato plants with or without sesame plants (Figure 6D, Table 1).

All plants grew and reached a similar size (Figure S4, treatment *F*_5,24_ = 1.164, *p* = 0.355 and date *F*_4,96_ = 73.704, *p* < 0.001,). Very few flowers were produced by the tomato plants during this experiment (e.g., no flowers in the no insect, whitefly only and *N. tenuis* whitefly with tomato and sesame plants treatments and ≤ 4 in the other treatments), therefore comparisons between the treatments were not made.

## 4. Discussion

The predatory and pestiferous status of a zoophytophagous mirid can vary in a cropping system [42,43]. Our field cage experiments provided a side by side evaluation of established zoophytophagous mirids to help determine which species may be most suitable for eventual use as biological control agent in integrated pest management (IPM) of cultivated tomatoes. This assessment is important as the benefit of plant feeding by zoophytophagous species must also be viewed in the rapid spatial and temporal changes that commonly occur in prey availability [43]. The balance in using a generalist species lies in having a plant predator that will readily attack and control the target pest (s), persist in the crop, and cause minimal damage.

The ability of *N. tenuis* to control whiteflies is well documented [22], and allowed comparison of native USA species to a known biological control agent. First described in Europe, *N. tenuis* has a worldwide distribution through human-assisted introductions and self-sustaining populations are found in Florida, California, Texas, Puerto Rico and New Mexico [44]. Although fortuitously established, concerns over the potential risk of injury to crops caused by *N. tenuis* feeding prompted the search for native mirid species that provided similar effective control of tomato pests and for ones that would also cause less damage. All three species reduced whiteflies numbers within two weeks of adding the mirids to infested plants and controlled their numbers over the six week evaluation. Despite being run several years apart under variable yearly weather conditions, these field cage studies were remarkably similar for the population growth of whiteflies and the impact of *M. praeclarus*. The ability of *M. praeclarus* and *E. modestus* to control whiteflies is not surprising given that both have been reported previously to feed on whitefly nymphs [32,34,37].

Although, all tested mirids controlled whiteflies, the damage they caused to tomato plants differed as well as their numerical response to the presence of the prey. As has been found in other studies [24,26], *N. tenuis* damaged tomato plants and the damage increased with time and as the whitefly numbers decreased. In contrast, *M. praeclarus* damage was minimal even in the near absence of prey. Interestingly, tomato plants with *M. praeclarus* had more flowers than all other treatments in both the 2015 and 2017 studies. In fact, there were more flowers found on tomato plants with *M. praeclarus* than on tomato plants with no insects (not mirids and no whiteflies). Mirid herbivory is known to induce defense responses in tomato plants [3,45] and this ability is species dependent [46]. Because different hormonal pathways may be up-or-down regulated due to the herbivory of *M. praeclarus* [38], further studies are warranted to explore whether this increase in flower number could be related to its herbivory and whether this could led to increased tomato production. Laboratory studies showed that *M. praeclarus* feeding did not cause damage rings or wilting of stems compared to *N. tenuis* [38]. *N. tenuis* and *M. praeclarus* showed a numerical response to prey with numbers of mirid nymphs being highest when whiteflies were most abundant. The number of *E. modestus* decreased 1.5 fold from the initial number added and the very few *E. modestus* caused damage to tomato plants and reduced flower production as previously reported [29]. The damage to tomato was very similar for *N. tenuis* and *E. modestus*. Both species feed mainly on the vascular bundles of tomato plants, a type of feeding uncommon among zoophytophagous mirids [4,29,47], which can lead to economic damage [4,5,16]. In contrast, *Macrolophus* species used in biocontrol programs are widely considered harmless [1]. However, *M. caliginosus* Wagner can damage tomatoes and cause economic loss through flower and fruit drop [48] and high densities of *M. pygmaeus* can cause significant tomato fruit damage, particularly when the plant was infected with Pepino mosaic virus [49,50]. As found with other *Macrolophus* biocontrol agents, careful management of *M. praeclarus* numbers may be necessary and further studies should be conducted.

There are benefits of phytophagy of plant predator mirids as it may help retain and increase numbers of the predator in a cropping system [28,51]. A mirid’s ability to survive in a crop environment in the absence of prey varies considerably among different mirid species on different crops [52,53,54]. Both *N. tenuis* and *M. praeclarus* have shown a preference for the odors of tomato over other crop plants [38,55]. However, *N. tenuis* was found to prefer the odors of sesame plants [56] and showed a preference to oviposit on sesame plants over tomato plants [28]. In the absence of prey, sesame plants were selected by *N. tenuis* over tomato plants in our field cage studies. *N. tenuis* numbers increased 13.5 fold and nymphs were able to reach the adult stage when sesame plants were present as has been previously reported [28,57]. In the absence of prey, sesame plants were selected by *N. tenuis* over tomato plants in our field cage studies. *N. tenuis* numbers increased 13.5 fold and nymphs were able to reach the adult stage when sesame plants were present as has been previously reported [28,57]. Interestingly, the large increase in *N. tenuis* numbers did not increase damage on tomato plants. In laboratory choice tests, the presence of sesame plants significantly reduced *N. tenuis* damage to tomato plants [28]. The six week duration of our study and the combined feeding of confined adults and nymphs resulted in similar damage to tomato plants, with or without sesame plants, but the damage in cages with sesame plants was not proportional to the number of *N. tenuis* in the cage.

*Macrolophus praeclarus* also benefited from the additional resources provided by the sesame plants. Significantly more nymphs and adults were able to survive six weeks in the absence of prey when sesame plants were present. However, *M. praeclarus* nymphs did not reach the adult stage as was seen with *N. tenuis* in this experiment. This has been found with other species within the genus *Macrolophus*. For example, *M*. *pygmaeus* did not complete development on tomato, pepper (*Capsicum annuum* L.), black nightshade (*Solanum nigrum* L.), and other host plants for Dicyphini species in the absence of *E. kuehniella* eggs [58]. In cultivated tomato, prey availability may fluctuate as not to allow mirids to persist. Further studies are needed to find a host plant that would be most suitable to attract and to maintain multiple plant predator populations in proximity of tomato crops [28,56].

The presence of the sesame plant did not change the ability of *N. tenuis* and *M. praeclarus* to control whitefly. However, the presence of the sesame plants increased the number of mirids. This was likely due to the combined benefit of the sesame plants and that these plants maintained a population of whiteflies. The presence of whiteflies on sesame plants may explain, in part, why there was significantly more *M. praeclarus* on sesame plants compared to tomato plants when whitefly prey was present compared to no differences in their location in the absence of prey. Interestingly, there were 95% fewer whiteflies on sesame plants in cages with *M. praeclarus* compared to sesame plants in cages with *N. tenuis*. The zoophytophagous predators’ potential to control pests on a companion crop may be an important aspect in determining the benefit of releasing one mirid over the others.

Our studies provided a mechanism to evaluate the ability of established and native mirids to control a pest species as well as aspects that would help retain the mirids near tomato plants and whether the mirids would cause damage to tomato plants. Two of the mirid species, *M. praeclarus* and *N. tenuis*, warrant further study and field testing. *M. praeclarus* had the benefits of responding numerically to whitefly infestation and caused minimal damage tomato relative to the other species tested. However, *M. praeclarus* may be harder to retain in the system in the absence of prey. The use of *N. tenuis* is well established but further work to utilize this biocontrol agent in open field tomato is needed particularly as populations of this species need to be carefully managed to prevent economic loss. Although *E. modestus* showed ability of control whiteflies, the inability of the mirid to increase numbers on prey, the high level of damage cause by few individuals, as well as the perception of growers of its pest status do not make it a favorable candidate for field release. Future studies of the interaction between *E. modestus* and the other mirid species should be explored. Specifically, the possibility that *N. tenuis* or *M. praeclarus* could be used to reduce *E. modestus* numbers in tomatoes through competition or intra-guild predation.

Most crops are attacked by more than one species of pests and biocontrol programs increasingly are utilizing generalist predators [59,60,61,62]. *M. praeclarus* and *N. tenuis* may provide a good option to control whiteflies, which is an immediate threat to tomato production. Further work to facilitate their establishment and retention in tomato plantings or in the environs of the field would also help growers prepare for imminent pest threats. Important lepidopteran pests such as *T. absoluta* and *H. armigera* are rapidly spreading and threaten tomato production systems in North America, the Caribbean region, and elsewhere [63,64]. *N. tenuis* has been shown to successfully switch from whiteflies to preying on *T. absoluta* soon after its arrival in the Mediterranean [65]. Field observations of *M. praeclarus’* predation on Old World bollworm and multiple other species suggest this mirid may also play an important role in a rapid response to potential invasion of new pest [36]. Additionally, further studies are needed to explore the interaction of these mirids with other biocontrol agents for whiteflies and other important pests of tomato.

## 5. Conclusions

The USA’s established and native mirid species could be immediately available to manage existing and future invasive pests on the tomato crop. However, these studies showed that not all generalist mirids would be suited for use as biocontrol agents for IPM of tomatoes. The native species, *M. praeclarus*, showed particular potential as this mirid caused very minimal damage to tomatoes and quickly controlled pest populations. Additionally, the use of alternative hosts to reduce damage to the tomato crop and retain the plant predator will likely vary for the species.

## Figures and Tables

**Table 1:** The result of the analysis of variance showing main effects and interactions on the total number of whitefly (*Bemisia tabaci* [*B.t.*]), mirids (*Macrolophus praeclarus* [*M.p.*], *Engytatus modestus* [*E.m.*] and *Nesidiocoris tenuis* [*N.t.*]), and mirid damage to tomato plants (Necrotic Rings).

Experiment	Source	Response Variable								
		Whiteflies			Mirids			Necrotic Rings		
		d.f.	*F*	*p*	d.f.	*F*	*p*	d.f.	*F*	*p*
*M.p.* vs. *E.m.*: Tomato + *B.t.*	Treatment	2, 11	15.71	<0.001	1, 7	13.50	0.008	1, 7	2.82	0.14
	Week	4, 44	5.46	<0.001	4, 28	0.99	0.43	4, 28	2.13	0.10
	Treatment × Week	8, 44	6.34	<0.001	4, 28	2.08	0.11	4, 28	1.75	0.17
*M.p.* vs. *N.t.*: Tomato + *B.t.*	Treatment	2, 15	17.38	<0.001	1, 10	1.15	0.29	1, 10	22.37	<0.001
	Week	4, 60	9.01	<0.001	4, 40	1.75	0.16	4, 40	26.41	<0.001
	Treatment × Week	8, 60	7.96	<0.001	4, 40	0.86	0.49	4, 40	5.48	<0.001
*M.p.* vs. *N.t.*: Tomato + Sesame + *B.t.*	Treatment	4, 20	20.57	<0.001	3, 16	2.72	0.08	3, 16	21.78	<0.001
	Week	4, 90	9.01	<0.001	4, 64	8.22	<0.001	4, 64	29.65	<0.001
	Treatment × Week	16, 90	7.16	<0.001	12, 64	2.07	0.03	12, 64	5.88	<0.001
*M.p.* only: Tomato + Sesame + *B.t.*	Treatment				1, 8	6.95	0.03	1, 8	0.20	0.88
	Week				4, 32	4.37	0.006	4, 32	1.61	0.19
	Treatment × Week				4, 32	0.35	0.87	4, 32	0.04	0.997
*N.t.* only: Tomato+ Sesame + *B.t.*	Treatment				1, 8	0.32	0.58	1, 8	0.17	0.69
	Week				4, 32	6.66	<0.001	4, 32	36.33	<0.001
	Treatment × Week				4, 32	3.50	0.02	4, 32	1.38	0.26

## Supplementary Materials

The following are available online at https://www.mdpi.com/2075-4450/11/10/670/s1, Figure S1: The daily high, average and low temperatures (°C) recorded over the course of the 4 experiments, Figure S2: The daily high, average and low relative humidity (RH) recorded over the course of the 4 experiments, Figure S3: Mean (±SE) height (cm) and number of flowers found on tomato plants in two five week field cage studies with three different mirid predators. Experiment 1 (A and C) compared plant growth in cages with clean plants (No Insects), whiteflies and no predators (*B. tabaci*), predator *Macrolophus praeclarus* + whitefly prey (*M. praeclarus* + *B. tabaci*) and predator *Engyatus modestus* and whitefly prey (*E. modestus* + *B. tabaci*). Experiment 2 (B and D) compared plant growth in cages with clean plants (No Insects), whitefly and no predators (*B. tabaci*), predator *Macrolophus praeclarus* and whitefly prey (*M. praeclarus* + *B. tabaci*) and predator *Nesidiocoris tenuis* and whitefly prey (*N. tenuis* + *B. tabaci*), Figure S4: Mean (±SE) height (cm) of tomato plants in five week field cage studies with clean plants (No Insects), whiteflies and no predators (*B. tabaci*), on whitefly infested tomato plants with *Macrolophus praeclarus* in cages with only tomato plants (*M. praeclarus* + *B. tabaci* + Tomato) or five tomato plants with one sesame plant added (*M. praeclarus* + *B. tabaci* + Tomato +Sesame) and on prey infested tomato plants with *Nesidiocoris tenuis* in cages with only tomato plants (*N. tenuis* + *B. tabaci* + Tomato) or five tomato plants with one sesame plant added (*N. tenuis* + *B. tabaci* + Tomato +Sesame), Data File S1: Supporting Field Cage Study Data (available upon request during review process and upon acceptance of manuscript).
