# Supplementary figures and images for: Biological Control Potential and Drawbacks of Three Zoophytophagous Mirid Predators against Bemisia tabaci in the United States
Source: Insects. 2020 Oct 1;11(10):670. doi: 10.3390/insects11100670 (PMC7600543; doi:10.3390/insects11100670)

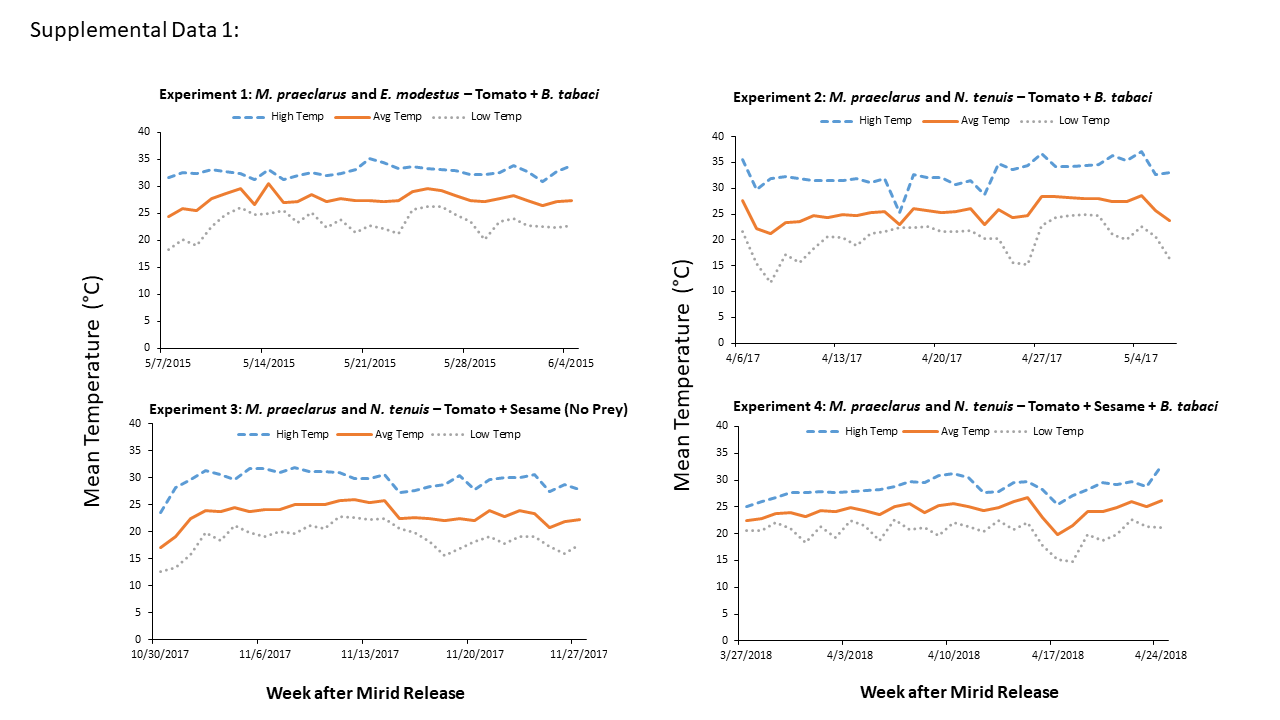

Supplement: Supplementary file 1 [file insects-11-00670-s001.zip › Supplemental Figures Roda et al/Supplemental Fig S1 Roda et al.TIF]

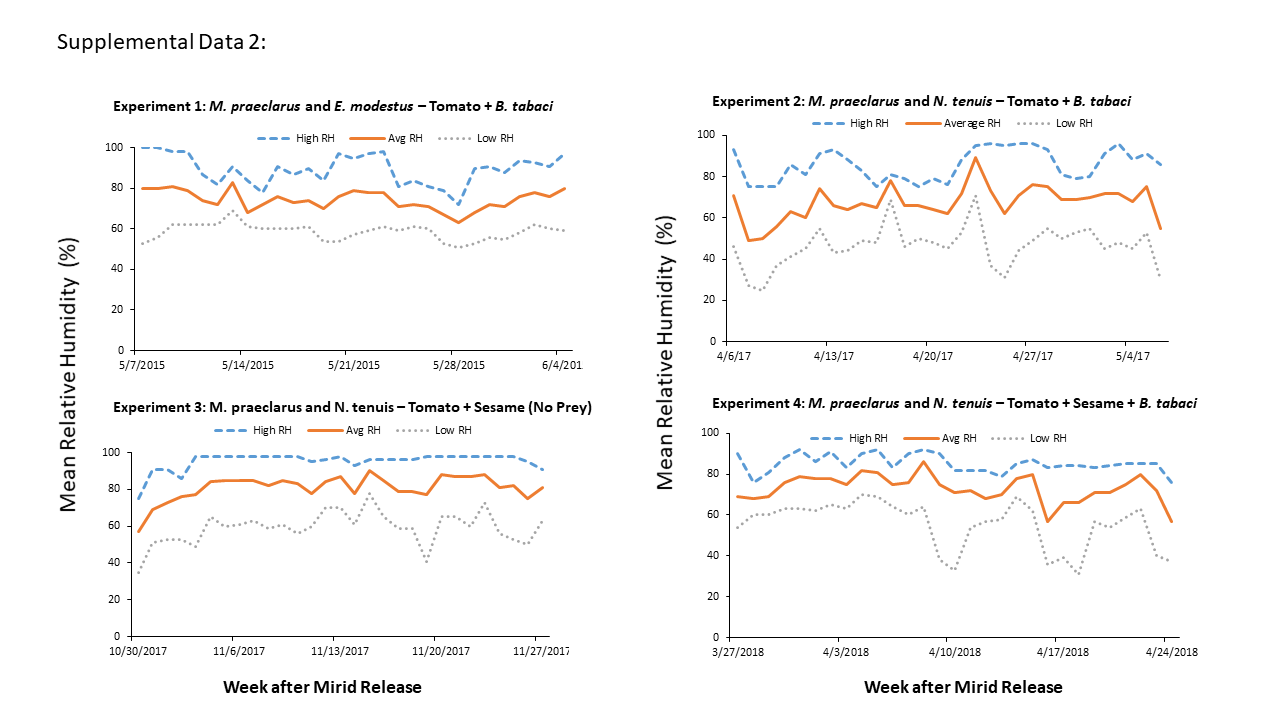

Supplement: Supplementary file 1 [file insects-11-00670-s001.zip › Supplemental Figures Roda et al/Supplemental Fig S2 Roda et al.TIF]

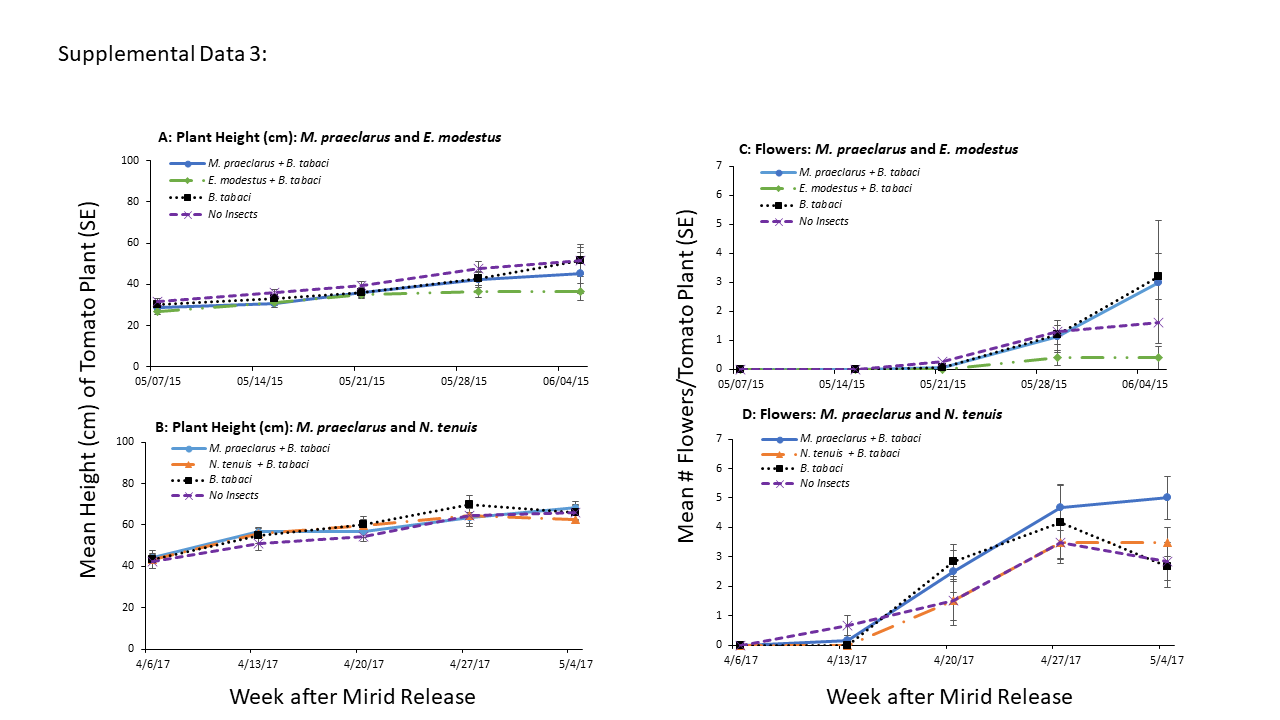

Supplement: Supplementary file 1 [file insects-11-00670-s001.zip › Supplemental Figures Roda et al/Supplemental Fig S3 Roda et al.TIF]

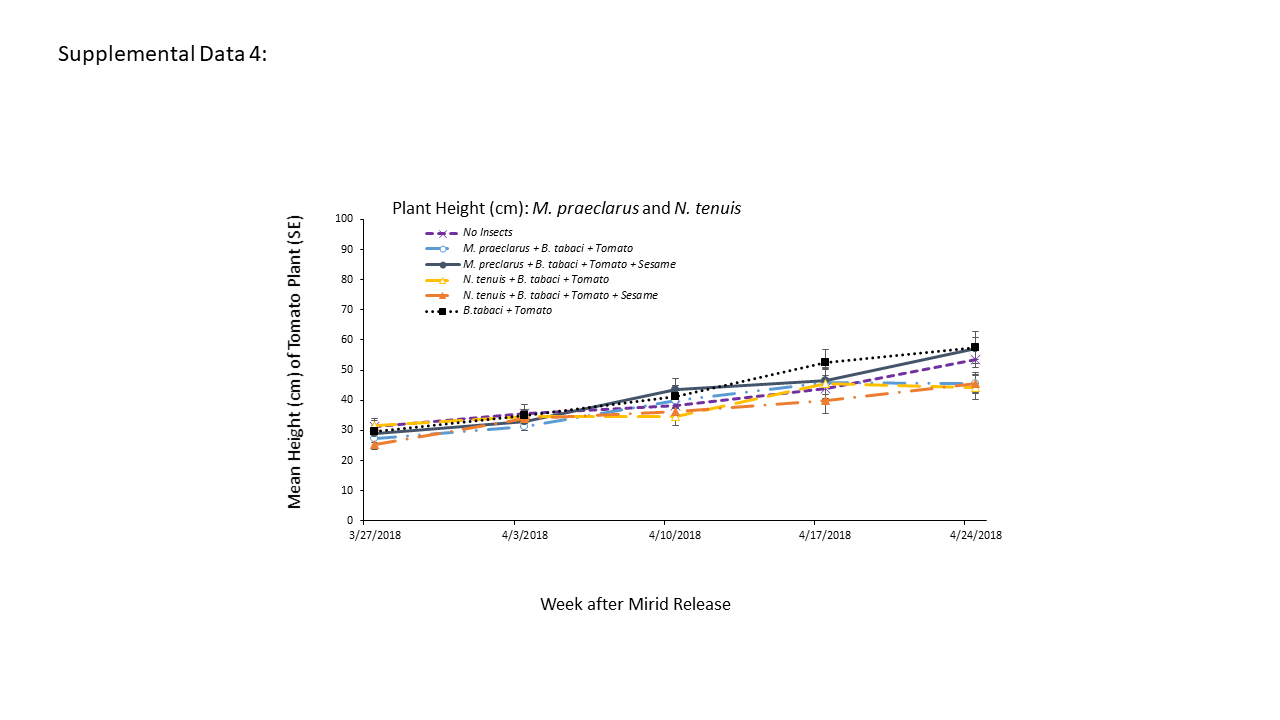

Supplement: Supplementary file 1 [file insects-11-00670-s001.zip › Supplemental Figures Roda et al/Supplemental Fig S4 Roda et al.TIF]
